# Supplementary figures and images for: Olfactory ensheathing cells from adult female rats are hybrid glia that promote neural repair
Source: eLife. 2025 Apr 29;13:RP95629. doi: 10.7554/eLife.95629 (PMC12040321; doi:10.7554/eLife.95629)

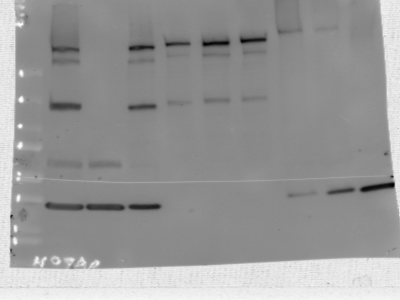

Supplement: Figure 9—source data 1. [file elife-95629-fig9-data1.zip › Figure 9eΓÇôSource Data unlabelled 1.tif]

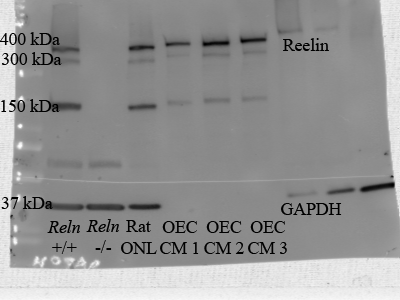

Supplement: Figure 9—source data 2. [file elife-95629-fig9-data2.zip › Figure 9eΓÇôSource Data 2 Labelled.tiff]

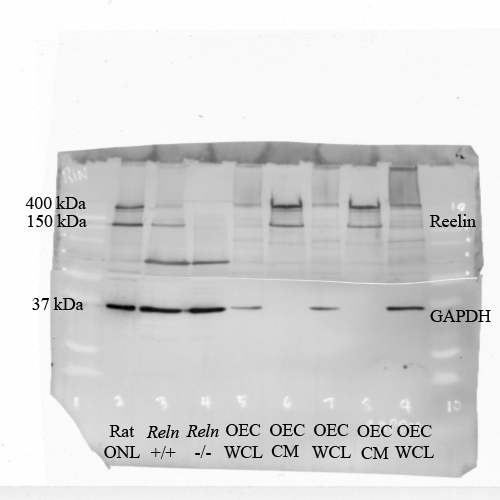

Supplement: Figure 9—source data 2. [file elife-95629-fig9-data2.zip › Figure 9ΓÇôSource Data 1 Labelled.tiff]
